# Feline *Leishmania* spp. Infection in a Non-Endemic Area of Northern Italy

**DOI:** 10.3390/ani10050817

**Published:** 2020-05-08

**Authors:** Eva Spada, Roberta Perego, Fabrizio Vitale, Federica Bruno, Germano Castelli, Gaia Tarantola, Luciana Baggiani, Sonia Magistrelli, Daniela Proverbio

**Affiliations:** 1 Dipartimento di Medicina Veterinaria (DIMEVET), Università degli Studi di Milan, via dell’Università 6, 26900 Lodi, Italy; gaiatarantola89@gmail.com (G.T.); luciana.baggiani@unimi.it (L.B.); daniela.proverbio@unimi.it (D.P.); 2 Centro di Referenza Nazionale per le Leishmaniosi (C.Re.Na.L), Istituto Zooprofilattico Sperimentale (IZS) della Sicilia A. Mirri, Via G. Marinuzzi 3, 90129 Palermo, Italy; fabrizio.vitale@izssicilia.it (F.V.); federicabruno4486@gmail.com (F.B.); germanocastelli@gmail.com (G.C.); 3 Agenzia di Tutela della Salute di Milano (Dipartimento Veterinario), Corso Italia 19, 20122 Milan, Italy; SMagistrelli@ats-milano.it

**Keywords:** epidemiology, feline, indirect immunofluorescence test, infection, leishmaniosis, Northern Italy, polymerase chain reaction, prevalence, stray cats

## Abstract

**Simple Summary:**

Leishmaniosis caused by *Leishmania infantum* is a parasitic disease of people and animals transmitted by sand fly vectors. Although dogs in south and central Italy are most affected, in recent decades feline leishmaniosis (FeL) has become an emerging disease. This study aimed to update information on the presence of FeL in stray cats in Milan, in northern Italy; to compare these results with previous studies performed in the same area; and to report aspects of *Leishmania* infection in cats. A total of 117 stray cats were included and 10 (8.6%) had *L. infantum* infection. The parasite was identified in popliteal lymph nodes in five cats and five had antibodies against *L. infantum.* Most infected cats were from a specific area of Milan. Increased gammaglobulins were seen in infected cats, which also had antibodies against the pathogens *Anaplasma phagocytophilum*, *Chlamydophila felis,* and *Toxoplasma gondii*. There was a high prevalence of FeL in the surveyed area of northern Italy. Further studies are needed to understand if these infected cats are being imported from other areas, or if there is a real *Leishmania* focus in Milan. Investigation of the presence of sand fly vectors in Milan would be useful.

**Abstract:**

Feline leishmaniosis (FeL) is an emerging vector-borne feline disease, with increasing numbers of cases reported and studies performed internationally. This study aimed to update the epidemiological status for FeL in stray cats in Milan, northern Italy; compare these results with previous studies in Northern Italy; and report clinicopathologic findings and coinfections in cats infected with *Leishmania* spp. A total of 117 cats were tested for *L. infantum* and retrovirus infection, hematological, and biochemical parameters. Demographic and clinical data were collected and FeL affected cats screened for selected coinfections. Overall, 10/117 (8.6%) cats tested positive for *L. infantum*: in five cats *L. infantum* DNA was found in popliteal lymph nodes and five were IFAT seropositive at titers from 1:80 to 1:160. Infected cats were concentrated in a specific area of Milan (*p* = 0.0154). No specific clinicopathologic abnormalities or retroviral infections were significantly linked to the infection, other than hypergammaglobulinemia (*p* = 0.0127). Seroreactivity to *Anaplasma phagocytophilum*, *Chlamydophila felis,* and *Toxoplasma gondii* was found in some infected cats. A high prevalence of FeL was found in a non-endemic area of northern Italy and future studies should continually monitor this data to understand whether these cases are imported or if *Leishmania* vectors are present in this area.

## 1. Introduction

Feline leishmaniosis (FeL) is an emerging feline disease, with an overall estimated prevalence of 10%. Cases are being more frequently reported and numerous international research studies have been published in recent years [1,2]. Cats are naturally infected by, and are susceptible to the same *Leishmania* species that affect dogs and people, but most cases involve *Leishmania infantum* as in the countries in the Mediterranean basin [3].

A high seroprevalence rate (12.2%) of FeL is found in Southern European countries including Italy, Spain, Portugal, and Greece. However in Italy, both the overall seroprevalence (24%) and polymerase chain reaction (PCR) prevalence (21%) were found to be higher than in other countries [2].

In endemic regions such as Mediterranean countries, the subclinical form of feline *L. infantum* is common, whereas clinical illness is relatively uncommon [1,2,4,5]. Sandflies are the natural vectors of *Leishmania* spp. and may also feed on the blood of cats. Infected cats may therefore be urban reservoirs of *Leishmania* spp. and transmit the protozoan to other sandflies [6]. In addition, cats can be considered sentinel reservoir hosts at least in endemic areas of zoonotic visceral leishmaniasis [2]. They can therefore play a role as additional reservoir hosts of *L. infantum* and, in a “One Health” perspective, preventative measures should be taken in this species based on epidemiological data.

Over recent decades, many studies have confirmed that feline *Leishmania* infection is relatively common in areas where canine leishmaniosis (CanL) is endemic. The Lombardy region (except for a limited focus) is not considered endemic for *L. infantum* infection at the time of writing. However, previous studies found a seroprevalence up to 12.2% in stray cats from Northern Italy [7,8].

The aims of this study were (I) to update the epidemiological status for *L. infantum* infection in stray cats from Milan, Northern Italy using indirect fluorescent antibody test (IFAT) on serum and real-time polymerase chain reaction (RT-PCR) assays applied to a variety of biological samples; (II) to compare results with recent studies performed in owned and stray cats from the same, and from different, areas of Northern Italy; and (III) to report clinicopathological findings and coinfections of cats infected with *L. infantum* and to compare demographic, physical, and clinicopathologic findings between infected and uninfected cats to find significant associations with infectious status.

## 2. Materials and Methods

### 2.1. Study Population and Sample Collection

Stray cats in Milan city (45°28′38”28 N, 09°10′53”40 E), the capital of the Lombardy region in Northern Italy, involved in a trap–neutered and release (TNR) program were prospectively randomly sampled with no limitation for age, gender, and clinical status between June 2016 and December 2018. The TNR program was part of a national program to control stray pet populations under Italian National Law (law no. 281/1991). The project underwent ethical review and was given approval number OPBA_108_2018 by the University of Milan Animal Welfare Bioethical Committee.

Data on signalment including: breed, gender, age (estimated based on dentition, animals <6 months of age were considered juvenile, whereas all others were considered adult) and colony of origin based on the nine municipalities of Milan city (zone 1–9) were collected for each cat.

During general anesthesia for neutering surgery the following clinical data were collected: rectal temperature; body condition score (BCS) evaluated by palpation of bone prominences and visual assessment of the animal’s silhouette with a score of 5/9 indicating normal BCS, 4–1/9 underweight, and 6–9/9 overweight [9]; data on clinical status based on physical and dermatological examination; cats were classified as either apparently healthy or unhealthy, when one or more of the following clinical abnormalities were present: fever, pale, or icteric mucous membranes, lymphadenopathy, cachexia, dehydration, gingivitis and/or stomatitis, emaciation, hepatomegaly, splenomegaly, dermal lesions, ocular lesions, signs of respiratory infections, and/or any other clinical findings indicating general illness.

The follow biological samples were collected: a 2.5 mL total volume blood sample collected by jugular or cephalic venipuncture into tubes with ethylenediaminetetraacetic acid (EDTA) anticoagulant (1 mL) and in plain collection tubes (1.5 mL) to obtain serum after centrifugation; fine needle aspirate from one popliteal lymph node; conjunctival swab from both eyes using sterile cotton swabs manufactured for bacteriological isolation, rubbed against the surface of the lower eyelid; dermal swabs from dermal lesions; and ectoparasites (ticks or fleas) when present.

### 2.2. Laboratory Analyses 

EDTA whole blood samples were examined within 12 h of collection for complete blood count (CBC). The remaining EDTA whole-blood samples, and oculoconjunctival and dermal swabs, and lymph node aspirates and ectoparasites were stored frozen at −20 °C and sent to the Experimental Zooprophylactic Institute (IZS) of Sicily, National Reference Centre for Leishmaniosis (C.Re.Na.L.), where RT-PCR analysis was performed to determine the presence of *Leishmania spp.* DNA. 

After centrifugation of blood collected in plain tubes one aliquot of serum sample was used to perform serology for retroviral infections and for selected coinfections, for measurements of serum protein content and for protein electrophoresis. An aliquot of each serum sample was stored frozen at −20 °C until sent to the IZS of Sicily where anti-*L. infantum* antibody titer was determined by immunofluorescence antibody test (IFAT).

#### 2.2.1. Hematological Parameters 

The following hematological parameters were assessed using an automated multiparameter hematology analyzer with software for animal samples (Cell-Dyn 3500 analyzer, Abbott, Rome, Italy): red blood cell (RBC) count, hemoglobin (Hb), hematocrit (HCT), mean cell volume (MCV), mean cell Hb (MCH), mean cell Hb concentration (MCHC), RBC distribution width (RDW), platelet (PLT) count, and white blood cell (WBC) count. Air-dried Wright-Giemsa-stained blood films were prepared (May-Grünwald-Giemsa MGG Quick Stain, Bio-Optica Milano Spa, Milan, Italy) and evaluated microscopically at oil immersion × 100 magnification (Motic^®^ BA 300, Milan, Italy) for the leukocyte differentiation (neutrophils, lymphocytes, eosinophils, basophils, and monocytes count) and the platelet count evaluation. 

#### 2.2.2. Serum Protein and Protein Electrophoresis 

Serum total protein concentration was measured by spectrophotometry using the colorimetric biuret method (Hagen Diagnostica S.R.L., Florence, Italy) on a Cobas Mira Classics Roche automated chemistry analyzer (Roche S.p.A., Mannheim, Germany). Albumin and protein fractions were analyzed with Hydragel Kit β1-β2 using a semiautomatic agarose gel electrophoresis system (Hydrasys, Sebia PN 1210, Issy-les-Moulineaux, France). Using the computer software Phoresis (Sebia PN 1210, Issy-les-Moulineaux, France) for Windows 2000 or XP Pro the electrophoretic curve for each sample was displayed. Protein fractions were determined based on the percentage of optical absorbance and the absolute concentration/dL was automatically calculated from the total serum protein concentration. Albumin to globulin (A/G) ratios were also calculated.

#### 2.2.3. Detection of *L. infantum* by IFAT

Specific antibodies to *L. infantum* (WHO strain: MHOM/IT/80/IPT1) were detected using the IFAT against in-house cultured promastigotes following Office International des Epizooties (OIE) Terrestrial Manual protocol [10]. Leishmania strain was used as an antigen fixed on multispot microscope slides (Bio-Merieux, Marcy L’Etoile, France) in an acetone bath. The feline sera were prepared by serial 2-fold dilutions (1:40 to 1:5120) in phosphate buffered saline (PBS), pH 7.2, and added to the antigen-coated wells. The slides were incubated for 30 min at 37 °C. Positive and negative controls were included in each series of analyzed samples. The positive control consisted of a known titer serum of a cat with a positive cultural isolation. The negative control consisted of serum from a cat testing negative to the culture. Fluoresceinated anti-cat immunoglobulin G (IgG) antibody (working anti-feline Anti-Cat IgG (whole molecule)—FITC antibody produced in goat, Sigma Aldrich, Saint Louis, MO, USA) was used (dilution 1:200 in PBS). The cut-off value for positivity was set at 1:80 according to the OIE Terrestrial Manual [10]. The slides were examined using a Leica DM 4000B fluorescence microscope (Leica, Heerbrugg, Switzerland). 

#### 2.2.4. Leishmania DNA Extraction and PCR Assays

EDTA whole-blood samples, oculoconjunctival swabs from both eyes, dermal swabs, ectoparasites, and popliteal lymph node aspirates were extracted by “PureLink^®^ Genomic DNA Mini Kit” (Invitrogen, Carlsbad, CA, USA) according to the manufacturer’s instructions. Lymph node aspirates, oculoconjunctival and dermal swabs, and ectoparasites were resuspended in phosphate buffer saline (PBS) solution (200 μL) and homogenized.

The RT-PCR was carried out in a CFX96 Real-time System (Bio-Rad Laboratories s.r.l., Hercules, CA, USA) using TaqMan Master Mix (Applied Biosystems by ThermoFisher, Waltham, MA, USA) and performed as previously described [11]. The target DNA for amplification is a 116-bp fragment in the constant region of the kDNA minicircle. This is one of the kDNA minicircle families currently used to identify the *Leishmania* genus. The primer sequences were: U 5′-GGCGTTCTGCGAAAACCG-3′; D5′-AAAATGGCATTTTCGGGCC-3′; while the associated probe was: 5′-TGGGTGCAGAAATCCCGTTCA- 3′ 5′FAM and 3′ black hole quencher (BHQ) labeled. The thermal cycling conditions comprised of an initial incubation for 2′ at 50 °C for uracil-N-glycosylase activity. This step was followed by a 0′ denaturation at 95 °C and 45 cycles at 95 °C for 15” and 60 °C for 1′ each. Samples were amplified in a single 96-well plate. On each plate, a negative control was included. Each standard, each sample, and the negative control were analyzed in triplicate for each run. Cycle threshold (Ct) value was calculated for each sample by determining the point of the fluorescence value exceeding the threshold limit. A positive control containing genomic *L. infantum* DNA and a negative control without DNA were included. The parasitic DNA load was determined in each examined sample by comparison of the data with a specific standard curve based on the number of *Leishmania* per milliliter of extracted volume. Standard curves were prepared for both the *Leishmania* gene target and the internal positive control (IPC Applied Biosystems, ThermoFisher, Waltham, MA, USA). A stock solution of *L. infantum* DNA was obtained by extraction from 10^9^ promastigotes/mL. Ten fold serial dilution of the DNA stock solution were performed to obtain the six points of the curve spanning from 10^6^ to 10^1^ DNA equivalent cells. The standard curve, calculated by independent experiments was linear over at least 6 log ranges of DNA concentration points with an average correlation coefficient of 0.988. The difference for each point of the curve was one log factor [11].

#### 2.2.5. Detection of Coinfections 

Serum from cats with *L. infantum* infection was subsequently tested for the IgG antibodies against *Rickettsia conorii*, *Ehrlichia canis*, *Anaplasma phagocytophilum, Chlamydophila felis*, IgG, and immunoglobulin M (IgM) against *Toxoplasma gondii* and for feline retrovirus status. 

IgG against *Rickettsia conorii*, *Ehrlichia canis*, and *Anaplasma phagocytophilum* were tested using IFAT canine commercial kits (Fluo Rickettsia conorii, Fluo Ehrlichia canis, Fluo Anaplasma ph Dog, Biopronix Product Line, Agrolabo Spa, Scarmagno, Turin, Italy.) The manufacturer’s protocol was followed for all serological tests, using anti-feline IgG ready to use (antibody concentration 7.5 µg/mL) instead of the canine ones provided by the kit. The samples were classified as positive if fluorescence was observed at a serum dilution of 1:64 or higher [12]. IgG specific to *Chlamydophila felis* and IgG and IgM to *Toxoplasma gondii* were detected using a commercially available IFAT kit (Fluo Chlamydophila felis, Fluo Toxoplasma gondii, Biopronix Product Line, Agrolabo Spa, Scarmagno, Turin, Italy). Titers ≥ 1:32 [13] and ≥ 1:64 [14] were considered seroreactive and, therefore, indicative of *C. felis* and *T. gondii* exposure, respectively. Positive and negative controls were included for each IFAT assay.

In all feline populations the presence of antibodies against p24 and gp40 antigens of feline immunodeficiency virus (FIV) and the presence of p27 antigen of feline leukemia virus infection (FeLV) were checked on serum samples using a commercial rapid enzyme-linked immunosorbent assay (ELISA) kit (SNAP^®^ Combo Plus FeLV Ag/FIV Ab, IDEXX Laboratories, Europe).

### 2.3. Statistical Analysis

The data was analyzed using standard descriptive statistics and reported as mean ± standard deviation (SD) or median and range, based on their distribution. Univariate analysis of categorical data using the Fisher’s exact test or χ^2^ analysis was performed to determine possible associations between *L. infantum* infection and the different variables collected. Differences between clinicopathological parameters in infected and uninfected cats were tested with a Student’s *t*-test or Mann–Whitney test according to whether or not the data were normally distributed. Associations were described using a probability (*p*) value < 0.05 as statistically significant. Statistical analysis was performed using a commercially available software program (MedCalc Statistical Software version 19.1.3, MedCalc Software bv, Ostend, Belgium; 2019).

## 3. Results and Discussion

A total of 117 cats were randomly sampled during the study period. Demographic, physical, clinical data, and retrovirus status of the whole feline population is reported in Table 1. The median age for all cats was 2 years (range 4 months–10 years, SD ± 2.26 years), rectal temperature was measured in 86/117 (73.5%) cats with a mean of 37.4 °C (range 35.0–39.3, SD ± 0.83 °C).

For various technical reasons (insufficient volume of samples or no sample collected), not all data were available for all 117 cats. All 117 cats were tested with RT-PCR on conjunctival swabs, 102/117 were tested by IFAT for *L. infantum*, 109/117 cats were RT-PCR tested on whole blood, 115/117 on lymph node aspirate, 13/117 on ectoparasites, and 2/117 on dermal swabs from dermal lesions. For the same reasons only 104/117 whole blood samples were examined for CBC and 72/117 serum samples for total protein and protein electrophoresis. 

Overall, 10 out of 117 stray cats sampled (8.6%) tested positive for *L. infantum* infection. *Leishmania* DNA was found in the popliteal lymph node aspirates of five cats. An additional five cats were IFAT seropositive with a titer of 1:160 (two cats) and 1:80 (three cats; Table 2 and Table 3). A further eight (7.8%) cats were IFAT seroreactive at a titer of 1:40, which is below the cut off of 1:80 considered positive for *L. infantum* infection in cats [4,10,15].

Table 3 shows the results of other recent epidemiological studies performed in cats in Northern Italy. The first two studies [8,16] relate to the same population of stray cats and were performed in previous years by the authors of this study. The last two studies [17,18] relate to owned cat populations from cats surveyed in cities located in Northern Italy.

Canine leishmaniosis has spread northwards in Italy in the last decades [19,20], but data on the epidemiology of CanL in the Lombardy region are scant and out of date, therefore difficult to compare with results of the current study. Cases of CanL are commonly diagnosed in the area where we performed our study, but the history of the affected dogs always reveals that they have visited or lived in areas endemic for CanL [21,22]. A canine serological survey on 313 dogs in a public animal shelter performed more than 15 years ago (2002–2003) in the urban area of Milan found a CanL seroprevalence of 3.4% [23]. Although the history of dogs in animal shelters is often unknown, some of these dogs may have come from areas that are endemic for *L. infantum* infection. However, it is unlikely that all *Leishmania* seropositive cats found in our study population were infected and imported from endemic areas. Therefore the results of this study highlight a high prevalence of FeL for a non-endemic area for *Leishmania* infection when compared to CanL, a prevalence similar to that found in cats in areas of Southern Italy endemic for CanL and human leishmaniosis [17] and similar to colony and shelter cats in central Italy in which recently a seroprevalence of 3.5% (at cut off ≥1:80) was found [24]. The result of the current study is not surprising in the light of previous studies performed by the same authors in colony stray cats from Milan city. Stray cats in Milan have been found to be IFAT seroreactive for *L. infantum* since sample collection starting in 2008 [7]. However the FeL seroprevalence in this feline population has changed substantially since the first two studies were conducted and almost halved with respect to one study [7] and reduced to a third compared to the last study (Table 3) [8].

In general, the most common diagnostic test used in epidemiological studies is IFAT, however identification of *Leishmania* amastigotes in aspirated samples of bone marrow, spleen, and lymph node is specific and considered the gold standard method for diagnosing FeL [2,25]. Therefore, PCR is recommended preferentially over other diagnostic tests, especially when blood samples and other clinical samples contain a low parasitic burden [26]. *Leishmania spp.* DNA was found for the first time in stray cats in Milan in 2014 [8]. The current study found twice the prevalence of cats harboring Leishmania DNA with respect to the previous study [8], i.e., five cats in which parasite DNA was identified in the reticoloendothelial system by RT-PCR, confirming that these cats were indeed infected, some with a high parasitic load such as cat n. 5, a young entire female that had 125 *Leishmania*/mL in the popliteal lymph node aspirate.

As in previous studies we analyzed only stray cats, which may act as amplifying hosts and sentinels for vector-borne infections, some of which represent important zoonosis [27]. Stray cats receive no prophylaxis against fleas, ticks, and sand flies, which are potential vector of infections such as rickettsiosis, anaplasmosis, ehrlichiosis, and hematic mycoplasmosis, which have been identified to have a high prevalence in previous studies performed in stray cat populations in Milan [16,28]. Since stray cats are exposed to all vector-borne infections present in this area they can give information as to the rate of these infections in a specific area. However, one of the problems with working with stray cat populations is the lack of anamnestic data that meant we could not exclude the possibility that the infected cats had been imported from other areas of Italy (or from other countries in southern Europe) where FeL is endemic. As previously described sporadic or rare occurrences of FeL in non-endemic areas can be the consequence of rehoming or moving cats, such as cases seen in Switzerland in cats imported from Spain [29,30].

Three RT-PCR-positive cats in this study had an IFAT titer <1:40, and one a titer of 1:40. Therefore, as previously shown [18], it seems that there was no agreement between the results obtained by IFAT and PCR. This is a common finding in cats from *L. infantum* endemic areas, where discordant results between serological and molecular techniques have been reported previously [7,24,31,32,33,34]. As previously hypothesized, the negative results obtained in the IFAT could reflect an ineffective immune system response or could be explained by the absence of antibody production during an early stage of the infection. For this reason, both direct and indirect techniques should be performed in epidemiological studies.

In comparison with other recent studies [17,18] performed in cats living in cities in Northern Italy (shown in Table 3), the rates of infection in cats surveyed varied widely. This may be due to the methodology used (IFAT versus PCR, type of samples analyzed with PCR), the geographic area of Northern Italy studied and the population under study (cat’s life style, owned versus stray cats). Both previous studies were performed on owned cats in Northern Italy and had a seroprevalence at IFAT with ≥1:80 cut off titer rates from 1.3% to 11.8% [17,18]. The last study [18] performed by Urbani et al. showed a very high seroprevalence (11.8%), more than double that found in the current study (4.9%), and this could due to the fact the feline population surveyed comes from a city closer to the *L. infantum* endemic foci of central Italy and that the Emilia-Romagna region has been considered to be an endemic area for CanL [20]. PCR detection in these two studies ranged from 0.3% to 0.7%, which is lower than the results of the current study (4.4%). These studies used different primers in their RT-PCR [35,36] and different sensitivity of primers used could explain these differences. In addition, in feline epidemiological investigations most studies were performed on EDTA-blood and some positive cats were possibly missed because blood is not the most sensitive tissue for the detection of *L. infantum* DNA in dogs or cats [4,37]. The presence of *Leishmania* DNA in lymph node aspirates was the most common site for PCR identification of infection in the current study and detected the highest prevalence of infection for molecular testing when compared to all studies performed in cats from Northern Italy.

For the first time in the current study ectoparasites collected from cats in Northern Italy were analyzed by RT-PCR for presence of *Leishmania* DNA. *L. infantum* DNA was found in ticks and fleas removed from cats living in areas where canine leishmaniosis is endemic [38,39,40]. This opens the debate about the epidemiological role of ticks in feline leishmaniosis. For this reason we tested the ectoparasites we found in our cat population, but none of the 13 samples (11 fleas and 2 ticks) tested positive for the presence of *Leishmania* DNA at RT-PCR. A limitation is that since no ectoparasite was found on the infected cats we lack information on potential sources of infection in our feline infected population. In addition, only a limited number of ectoparasites were collected and analyzed.

The only statistically significant demographic factor linked to *L. infantum* infected cats was the origin from a colony located in area 9 of Milan city (*p* = 0.0154; Table 1). The fact that significantly more infected cats come from a specific area of Milan could be helpful to better understand whether there is a real endemic *Leishmania* focus in the city. For example, it may be useful to perform an entomological survey in this area to identify whether the classical vector of *Leishmania* spp., i.e., phlebotomine sand flies of genus *Phlebotomus* are present here. Until July 2019 there was no data on the presence of phlebotomine sand flies in the Milan area [41]. Sandflies included *Phlebotomus perniciousus* have previously been collected in the Lombardy region [20,41,42,43]. However, to the author’s knowledge, no sandflies have been identified in the area of Milan from which the cats in this study originated.

Clinical data, results of CBC and protein profile related to the 10 infected cats are reported in Table 4, Table 5, and Table 6, respectively.

Most infected cats showed solitary or generalized lymphadenomegaly and gingivostomatitis was the second most common clinical sign in the infected cats. When clinical signs of FeL are present, they are non-specific and frequently similar to those in other feline diseases and subclinical *L. infantum* feline infection is common [1,2,4,5,25,26,44,45]. The remaining subjects, 3/10 infected cats showed no clinical abnormalities and seemed to be apparently healthy and this corroborates the reports that FeL infected cats can be asymptomatic and that subclinical feline infection *L. infantum* is common [1,2,4,5,25,26,44,45,46,47].

Mild to severe CBC abnormalities such as normocytic normochromic non-regenerative anemia, moderate to severe pancytopenia or leucocytosis may be seen in *L. infantum* infected cats [1,4,5,30,48,49]. Most infected cats in the current study had CBC parameters outside the reference range for healthy cats, however no significant statistical difference was reported in CBC median value between infected and uninfected cats.

While hyperproteinemia results were not significantly different between infected and uninfected cats in our population, statistically significant differences were found in protein fractions at serum electrophoresis. A statistically significant lower and higher concentration was found in alpha 2-globulins and gamma-globulins in infected compared to uninfected cats, respectively, as shown in Table 7. While median alpha 2-globulins remain in reference range in FeL infected cats, median gamma-globulins increased more in infected than in uninfected cats. Gammopathy are found in most cases described in the literature [1,4,5,18,30,48,49,50,51,52] with some cats showing also a normal proteinemia as in the feline population analyzed in this study. It can be hypothesized that in cats, as in dogs, the humoral immune response could also be activated by the production of immunoglobulins after infection, resulting in high concentrations of gamma-globulins. However polyclonal gammopathy occurs in many feline infectious and inflammatory diseases and is not specific for FeL. In addition, we should consider that both clinical, CBC and serum protein electrophoresis results may have been influenced by concurrent disease as well as retroviral or other non-diagnosed infections, which are common in stray feline populations. This could be supported by the fact that uninfected cats in our population also showed a median gamma-globulins content above the reference range for healthy cats.

Some *L. infantum* infected cats were also retrovirus coinfected and seropositive for the presence of antibodies (IgG) to *Anaplasma phagocytophilum* and *Toxoplasma gondii*. All tested cats were seropositive for *Chlamydophila felis* as shown in IFAT results reported in Table 8. The prevalence of feline retroviruses in stray cats from our study area was slightly higher than rates detected in a previous study, 14.7% of the current study versus 10.4% in [53], and this was due to an increase in FeLV infections. This result was not surprising, as stray cats in Northern Italy are not routinely vaccinated against this retrovirus. A significant association between FIV and *L. infantum* infections has been found in previous studies [17,34,51,54] and also in stray cat populations in Milan investigated in a previous study [7]. FIV was previously reported to be the most frequent concomitant coinfection in FeL infected cats [1,5]. However, this significant association was not seen in the current study. In fact, although associations have been noted, immunosuppression induced by agents such as FIV does not always increase the risk for FeL, as in many studies there was no link between FIV infection and FeL infection [25,29,30,44,47,49,50,52,55,56,57].

FeL infection does not exclude the possibility of concurrent diseases or coinfections. In previous studies the association between coronavirus, Toxoplasma and some vector-borne coinfections in cats by antibody and/or PCR positive to *L. infantum* has been explored [8,31,34,56,57]. Coinfections or comorbidities are frequently detected in stray cats [8,31]. The presence of one or more concomitant infection may influence the clinical presentation and presence of laboratory abnormalities, can contribute to a misrepresentation of clinical FeL, influence parasite burden or alter the progression of FeL.

## 4. Conclusions

The results of the present study highlight a stable FeL situation among the stray cats of the Milan city where the cats are exposed to, or infected by *L. infantum*. As stray cats do not receive any prophylactic measure other than neutering surgery and many unhealthy stray cats are hospitalized, FeL should be considered in the differential diagnosis list for cats showing hypergammabulinemia of unknown origin. Future studies on FeL infection in the same area of Northern Italy are needed to clarify whether FeL cases are the result of rehoming or movement of cats from *L. infantum* endemic areas or whether there is a real FeL focus in this region. Continual monitoring and further studies to investigate whether *Leishmania* vectors are present in this area are required.

## Figures and Tables

**Table 1:** Demographic, physical, clinical data, and retroviral status of whole stray cat population, with both *Leishmania infantum* uninfected and infected cats. Due to insufficient volume of samples, only 95 cat samples were tested for of feline immunodeficiency virus (FIV) and feline leukemia virus infection (FeLV) infections.

Variables	Modalities	Whole Population (*n* = 117)	*Leishmania infantum* Infection Status		*p* Value
			Uninfected (*n* = 107)	Infected (*n* = 10)	
**Age**	Young	30 (25.6%)	25 (23.4%)	5 (50.0%)	0.0667
	Adult	87 (74.4%)	82 (76.6%)	5 (50.0%)	
**Gender**	Male	47 (40.2%)	40 (37.4%)	7 (70.0%)	0.0869
	Female	70 (59.8%)	67 (62.6%)	3 (30.0%)	
**Reproductive status**	Neutered	12 (10.3%)	10 (9.3%)	2 (20.0%)	0.2722
	Not-neutered	105 (89.7%)	97 (90.7%)	8 (80.0%)	
**Breed**	DSH	116 (99.1%)	106 (99.1%)	10 (100.0%)	1.0000
	Mixed Chartreux	1 (0.9%)	1 (0.93%)	0 (0.0%)	
**Origin**	Zone 1	12 (10.3%)	10 (9.3%)	2 (20.0%)	0.2722
	Zone 2	1 (0.9%)	1 (0.93%)	0 (0.0%)	1.0000
	Zone 3	2 (1.7%)	1 (0.93%)	1 (10.0%)	0.1643
	Zone 4	18 (15.4%)	18 (16.8%)	0 (0.0%)	1.0000
	Zone 5	17 (14.5%)	16 (15.0%)	1 (10.0%)	1.0000
	Zone 6	13 (11.1%)	12 (11.2%)	1 (10.0%)	1.0000
	Zone 7	27 (23.1%)	26 (24.3%)	1 (10.0%)	0.4499
	Zone 8	14 (12.0%)	13 (12.1%)	1 (10.0%)	1.0000
	Zone 9	7 (6.0%)	4 (3.7%)	3 (30.0%)	**0.0154**
	nr	6 (5.1%)	6 (5.6%)	0 (0.0%)	-
**Body condition score**	Normal	62 (53.0%)	56 (52.3%)	6 (60.0%)	0.7476
	Underweight	52 (44.4%)	49 (45.8%)	3 (30.0%)	0.5086
	Overweight	3 (2.6%)	2 (1.9%)	1 (10.0%)	0.2369
**Health status**	Healthy	97 (82.0%)	91 (85.0%)	6 (60.0%)	0.0665
	Unhealthy	20 (17.1%)	16 (15.0%)	4 (40.0%)	
**Ectoparasites**	Fleas	30 (25.6%)	29 (27.1%)	1 (10.0%)	0.4490
	Ticks	3 (2.6%)	3 (2.8%)	0 (0.0%)	1.0000
	Ear mites	8 (6.8%)	8 (7.5%)	0 (0.0%)	1.0000
**Retrovirus status** (*n* = 95)	Seropositive	14 (14.7%)	12/86 (14.0%)	2/9 (22.2%)	0.6167
	Seronegative	81 (85.3%)	74/86 (86.0%)	7/9 (77.8%)	
**Retrovirus infection** (*n* = 95)	FIV	6 (6.3%)	6/86 (7.0%)	0/9 (0.0%)	1.0000
	FeLV	6 (6.3%)	5/86 (5.8%)	1/9 (11.1%)	0.4590
	FIV+FeLV	2 (2.1%)	1/86 (1.2%)	1/9 (11.1%)	0.1814

DSH: domestic shorthair cat; nr: not reported; FIV: feline immunodeficiency virus; FeLV: feline leukemia virus. Results in bold indicate statistical significant *p* value < 0.05 at Fisher’s exact test or χ2 analysis.

**Table 2:** Signalment and results of real-time polymerase chain reaction (RT-PCR) and immunofluorescence antibody test (IFAT) for *L. infantum* in the 10 infected domestic shorthair cat (DSH) stray cats.

***n.***	Gender	Age (Years)	RT-PCR			IFAT Cut Off ≥ 1:80
			Whole Blood	Popliteal Lymph Node	Conjunctival Swab	
1	**F**	**2**	negative	**5 *Leishmania*/mL**	negative	1:40
2	NF	4	negative	**20 *Leishmania*/mL**	negative	<1:40
3	M	1	negative	**13 *Leishmania*/mL**	negative	<1:40
4	M	1	negative	**5 *Leishmania*/mL**	negative	<1:40
5	F	0.6	negative	**125 *Leishmania*/Ll**	negative	not tested
6	M	8	negative	negative	negative	**1:160**
7	M	1	negative	negative	negative	**1:160**
8	M	2	negative	negative	negative	**1:80**
9	NM	3	negative	negative	negative	**1:80**
10	M	1.1	negative	negative	negative	**1:80**

F: female; NF: neutered female; M: male; NM: neutered male; Positive results of RT-PCR and IFAT for *L. infantum* in bold.

**Table 3:** Comparison of results of epidemiological studies for *L. infantum* infection of the current study with other recent studies performed in cats in northern Italy.

Variable		Spada et al 2014 [7]	Spada et al 2016 [8]	Current Study	Iatta et al 2019 [17]	Urbani et al 2020 [18]
**Population**		Stray	Stray	Stray	Owned	Owned
**Geographic area**		Milan	Milan	Milan	Northern Italy	Bologna
**Sampling years**		2008-2010	2014	2016-2018	2017-2018	2017
**Samples number**		233	90	117	1543	152
**FeL overall prevalence**		9.0%	12.2%	8.6%	1.6%	12.5%
**IFAT overall seropositivity** **(at ≥1:80 cut off)**		21/233 (9.0%)	11/90 (12.2%)	5/102 (4.9%)	20/1543 (1.3%)	18/152 (11.8%)
**IFAT titer**	**1:40**	38 (16.3%)	16 (17.8%)	8/102 (7.8%)	0 (0.0%)	27 (17.8%)
	**1:80**	15 (6.4%)	11 (12.2%)	3/102 (2.9%)	20 (1.3%)	11 (7.2%)
	**1:160**	6 (2.6%)	0 (0.0%)	2/102 (2.0%)	0 (0.0%)	5 (3.3%)
	**1:320**	0 (0.0%)	0 (0.0%)	0/102 (0.0%)	0 (0.0%)	2 (1.3%)
**PCR overall positivity**		0 (0.0%)	2 (2.2%)	5/115 (4.4%)	5/1543 (0.3%)	1/150 (0.7%)
**PCR on**	**Whole blood**	0 (0.0%)	1 (1.1%)	0/109 (0.0%)	5 (0.3%)	0/146 (0.0%)
	**Conjunctiva**	np	0 (0.0%)	0/117 (0.0%)	np	0/150 (0.0%)
	**Lymph node**	np	1 (1.1%)	5/115 (4.4%)	np	np
	**Ectoparasites**	np	np	0/13 (0.0%)	np	np
	**Dermal lesion**	np	np	0/2 (0.0%)	np	np
	**Hair**	np	np	np	np	1/150 (0.7%)

FeL: Feline leishmaniosis; IFAT: immunofluorescence antibody test; PCR: polymerase chain reaction; np: not performed.

**Table 4:** Clinical data from ten *Leishmania infantum* infected stray cats from Northern Italy.

*n.*	Rectal Temperature (°C)	Mucous Membrane	Lymphadenomegaly	Respiratory Signs	Gastrointestinal Signs	Dernal Signs	Ocular Signs
1	38.4	n	np	np	np	np	np
2	37.7	n	Solitary	np	np	Bilateral temporal skin wounds	np
3	36.0	n	Solitary	np	Gingivostomatitis	np	np
4	37.4	n	Solitary	np	np	np	np
5	37.3	Pallor	np	np	Gingivostomatitis	np	np
6	37.8	Pallor	Solitary	Respiratory pulmonary rumors	Gingivostomatitis	np	Corneal ulcer and third eyelid lesion
7	nr	n	np	np	np	np	np
8	38.6	Pallor, subicterus	Generalyzed	np	np	np	np
9	nr	n	Solitary	np	np	Generalised scaling	np
10	37.4	n	np	np	np	np	np

BCS: body condition score; n: normal; np: not present.

**Table 5:** Complete blood count values in *L. infantum* infected stray cats from Northern Italy.

*n.*	RBCs (6560–11200 ×10^3^/μL)	Hb (10.6–15.6 g/dL)	Hct (31.7–48.0%)	MCV (36.7–53.7 fl)	MCH (12.3–17.3 pg)	MCHC (30.1–35.6 g/dL)	RDW (16.7–22.9%)	PLTs (175.0–500.0 ×10^3^/μL)	WBCs (4040–18700/μL)	Neutrophils (2300–14000/μL)	Lymphocytes (800–6100/μL)	Monocytes (0–700/μL)	Eosinophils (0–1500/μL)	Basophils (0–100/μL)
1	**5990**	**10.0**	**28.5**	47.5	16.6	35	**15.2**	470	13600	11696	1088	408	408	0
2	7080	**9.3**	**29.8**	42.0	13.1	31.2	17.8	397	**19500**	**14430**	2340	390	**2340**	0
3	8370	11.6	42.2	50.0	13.8	**27.5**	19.2	321	8600	6708	**516**	172	1204	0
4	8010	12.1	38.6	48.0	15.1	31.3	17.3	444	12900	9159	2580	129	1032	0
5	nd	nd	nd	nd	nd	nd	nd	nd	nd	nd	nd	nd	nd	nd
6	**5470**	**8.9**	**29.0**	**60.0**	16.2	**27.2**	**16.2**	393	**19100**	12033	4775	**1719**	573	0
7	8230	11.4	38.6	47.0	13.9	**29.6**	17.4	197	**25400**	**20066**	2286	**1016**	**1778**	**254**
8	8360	10.2	**29.4**	**35.2**	12.3	34.8	18.4	193	8580	5663	2574	0	343	0
9	8290	10.6	32.9	39.7	12.8	32.3	**29.7**	**537**	8250	5693	1485	577	495	0
10	8660	15.0	44.0	51.0	**17.4**	34.3	18.2	249	13800	10350	1656	276	**1518**	0

RBCs = red blood cells; Hb = hemoglobin; Hct = hematocrit; MCV = mean cell volume; MCH = mean cell hemoglobin; MCHC = mean cell hemoglobin concentration; RDW = red blood cell distribution width; WBCs = white blood cells; PLTs = platelets. In bold value outside the reference ranges; nd: not determined.

**Table 6:** Serum total protein, albumin and globulin content, and albumin to globulin ratio in *L. infantum* infected stray cats of Northern Italy.

*n*	Total Protein (6–8 g/dL)	Albumin (3.0–4.6 g/dL)	Alpha 1-globulins (0.1–0.5 g/dL)	Alpha 2-globulins (0.3–1.2 g/dL)	Beta 1-globulins (0.1–0.7 g/dL)	Beta 2-globulins (0.2–0.8 g/dL)	Gamma-globulins (0.3–0.8 g/dL)	A/G Ratio (0.8–1.6)
1	6.97	**2.91**	0.24	1.14	0.35	0.28	**2.06**	**0.72**
2	6.63	**2.96**	0.16	1.01	0.23	0.4	**1.87**	0.81
3	**5.64**	3.19	0.12	1.03	0.28	**0.16**	**0.86**	1.3
4	**5.59**	3.05	0.10	0.34	0.41	0.27	**1.41**	1.2
5	nd	nd	nd	nd	nd	nd	nd	nd
6	6.16	**1.94**	**0.09**	0.75	0.30	0.28	**2.8**	**0.46**
7	6.69	3.02	0.19	**1.4**	0.37	0.22	**1.49**	0.82
8	**11.93**	4.40	0.31	0.98	**0.73**	0.75	**4.76**	**0.58**
9	6.27	**2.95**	0.19	1.19	0.26	0.22	**1.47**	0.89
10	nd	nd	nd	nd	nd	nd	nd	nd

nd: not determined: A/G: albumin to glubulin ratio. In bold values outside the reference ranges.

**Table 7:** Comparison of total protein, albumin, globulin content, and albumin to globulin ratio between *L. infantum* infected and uninfected cats.

Parameter	*Leishmania infantum* Infection Status						*p* Value
	Infected Cats			Uninfected Cats			
	*n*	Median	Average Rank	*n*	Median	Average Rank	
**Total protein (6–8 g/dL)**	8	6.4	45.9	64	5.9	35.3	0.1761
**Albumin (3.0–4.6 g/dL)**	8	2.9	49.0	64	2.7	34.9	0.0731
**Alpha 1-globulins (0.1–0.5 g/dL)**	8	0.1	24.7	64	0.2	37.9	0.0914
**Alpha 2-globulins (0.3–1.2 g/dL)**	8	1.0	21.6	64	1.2	38.3	**0.0337**
**Beta 1-globulins (0.1–0.7 g/dL)**	8	0.3	29.0	64	0.3	37.4	0.2818
**Beta 2-globulins (0.2–0.8 g/dL)**	8	0.2	38.6	64	0.2	36.2	0.7535
**Gamma-globulins (0.3–0.8 g/dL)**	8	1.6	53.8	64	1.0	34.3	**0.0127**
**A/G ratio (0.8–1.6)**	8	0.8	34.5	64	0.8	36.7	0.7743

A/G: albumin to globulin ratio. In bold where statistical significant *p* value <0.05 at Mann–Whitney test.

**Table 8:** Coinfections, retrovirus status and seroreactivity to immunofluorescence antibody test in *L. infantum* infected stray cats in Northern Italy. Due to insufficient sample volume the end point titer for *Chlamydophila felis* was not determined.

***n.***	*Anaplasma phagocytophilum* (Cut off 1:64)	*Ehrlichia canis* (Cut off 1:64)	*Rickettsia conorii* (Cut off 1:64)	*Toxoplasma gondii*		*Chlamydophila felis* (Cut off 1:32)	FIV Ab	FeLV Ag
				IgG (Cut off 1:64)	IgM (Cut off 1:64)			
1	negative	negative	negative	negative	negative	**1:40**	negative	negative
2	negative	negative	negative	negative	negative	**1:80**	negative	negative
3	negative	negative	negative	negative	negative	**1:40**	negative	negative
4	negative	negative	negative	negative	negative	**>1:320**	negative	negative
6	negative	negative	negative	**1:128**	negative	**1:40**	**positive**	**positive**
7	negative	negative	negative	negative	negative	**1:40**	negative	**positive**
8	**1:64**	negative	negative	negative	negative	**>1:320**	negative	negative
9	negative	negative	negative	negative	negative	**>1:320**	negative	negative

nd: not determined; IgG: immunoglobulin G; IgM: immunoglobulin M; FIV Ab: feline immunodeficiency virus antibodies; FeLV Ag: feline leukemia virus antigen. In bold positive results.
